# Radiation pneumonitis in lung cancer patients treated with chemoradiation plus durvalumab

**DOI:** 10.1002/cam4.3113

**Published:** 2020-05-06

**Authors:** Narek Shaverdian, Maria Thor, Annemarie F. Shepherd, Michael D. Offin, Andrew Jackson, Abraham J. Wu, Daphna Y. Gelblum, Ellen D. Yorke, Charles B. Simone, Jamie E. Chaft, Matthew D. Hellmann, Daniel R. Gomez, Andreas Rimner, Joseph O. Deasy

**Affiliations:** ^1^ Department of Radiation Oncology Memorial Sloan Kettering Cancer Center New York New York USA; ^2^ Department of Medical Physics Memorial Sloan Kettering Cancer Center New York New York USA; ^3^ Thoracic Oncology Service Division of Solid Tumor Oncology Department of Medicine Memorial Sloan Kettering Cancer Center New York New York USA

**Keywords:** durvalumab, non–small cell lung cancer, radiation pneumonitis

## Abstract

**Introduction:**

Durvalumab after concurrent chemoradiation (cCRT) is now standard of care for unresected stage III non–small cell lung cancer (NSCLC). However, there is limited data on radiation pneumonitis (RP) with this regimen. Therefore, we assessed RP and evaluated previously validated toxicity models in predicting for RP in patients treated with cCRT and durvalumab.

**Methods:**

Patients treated with cCRT and ≥ 1 dose of durvalumab were evaluated to identify cases of ≥ grade 2 RP. The validity of previously published RP models was assessed in this cohort as well a reference cohort treated with cCRT alone. The timing and incidence of RP was compared between cohorts.

**Results:**

In total, 11 (18%) of the 62 patients who received cCRT and durvalumab developed ≥ grade 2 RP a median of 3.4 months after cCRT. The onset of RP among patients treated with cCRT and durvalumab was significantly longer vs the reference cohort (3.4 vs 2.1 months; *P* = .01). Numerically more patients treated with cCRT and durvalumab developed RP than patients in the reference cohort (18% vs 9%, *P* = .09). Among patients treated with cCRT and durvalumab, 82% (n = 9) were responsive to treatment with high‐dose glucocorticoids. Previously published RP models widely underestimated the rate of RP in patients treated with cCRT and durvalumab [AUC ~ 0.50; *p*(Hosmer‐Lemeshow): 0.98‐1.00].

**Conclusions:**

Our data suggest a delayed onset of RP in patients treated with cCRT and durvalumab vs cCRT alone, and for RP to develop in a greater number of patients treated with cCRT and durvalumab. Previously published RP models significantly underestimate the rate of symptomatic RP among patients treated with cCRT and durvalumab.

## Graphical abstract

Radiation pneumonitis (RP) is a significant toxicity of thoracic radiation, yet little is known regarding RP in lung cancer patients treated with definitive chemoradiation (cCRT) and durvalumab. We found patients treated with cCRT and durvalumab to have a significantly later onset of RP and a greater frequency of RP compared to patients treated with cCRT alone, we also found previously published toxicity models to be unsuccessful in predicting for RP among patients treated with cCRT and durvalumab.

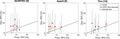

## 1 INTRODUCTION

The use of consolidative durvalumab, an antiprogrammed death ligand 1 (PD‐Ll) inhibitor, after concurrent chemoradiation (cCRT) has significantly improved patient survival and now represents the current standard of care for the treatment of unresected stage III non–small cell lung cancer (NSCLC).^1^ However, there is limited data on radiation pneumonitis (RP) with this regimen. Pneumonitis is a major dose‐limiting toxicity of both thoracic radiation therapy and of durvalumab.^2^, ^3^ The pathogenesis of RP is thought to be in‐part immune mediated and secondary to inflammatory cytokines.^4^, ^5^ Data from prospective studies have suggested increased pulmonary toxicity in patients who receive thoracic radiation with concurrent or sequential PD‐1/PD‐L1 therapies, suggesting a potential interaction between these therapies that may increase the risk of RP.^6^, ^7^

Multiple validated models have been developed to predict the likelihood of a patient developing RP.^8^, ^9^, ^10^ These toxicity models are used in everyday practice to optimize radiation treatment plans to minimize the risk of symptomatic RP. However, these models have all been derived from patients treated prior to the use of consolidative durvalumab. These models uniformly include the mean lung radiation dose (MLD) and may also include patient characteristics such as smoking history and preexisting pulmonary disease. To date, the validity of using these models to predict for symptomatic RP among patients treated with cCRT and consolidative durvalumab remains unknown. We, therefore, evaluated stage III NSCLC patients treated with cCRT and consolidative durvalumab to characterize the onset, imaging characteristics and clinical course of RP, and to test the accuracy of published models in predicting for symptomatic RP in patients treated with this new standard of care.

## 2 METHODS

### 2.1 Patients and treatment

Eligible patients had AJCC 8th edition stage III NSCLC and received curative intent cCRT and at least one cycle of durvalumab. The prescription dose ranged from 54Gy to 66Gy and was delivered in 2 Gy fractions using intensity‐modulated radiation therapy. Patients underwent free‐breathing 4‐dimensional computed tomography (CT) simulation for treatment planning on which the gross tumor volume was contoured. Patients had a diagnostic positron emission tomography scan available to guide target delineation. An internal target volume margin was added based on respiration motion, to which 5‐7 mm and 5 mm margins were added to account for microscopic disease and day‐to‐day setup uncertainty, respectively. Patients were treated with at least two cycles of platinum‐based chemotherapy concurrently with radiation therapy. Patients were evaluated for consolidative durvalumab after completion of cCRT. Patients without disease progression or persistent chemoradiation toxicity started durvalumab (10 mg/kg every 2 weeks) for up to 12 months of total consolidative therapy.

### 2.2 Follow‐up and radiation pneumonitis characterization

The clinical follow‐up schedule followed standard procedure and included history, physical, and chest CT every 3 months for the first 2 years. Patients were defined to have RP based off clinical and imaging findings. To be considered to have RP, patients met the following criteria: (a) had pulmonary symptoms including dyspnea and/or cough, (b) had CT‐based imaging changes involving the radiated field, and (c) had symptoms occur within 12 months from the completion of radiation therapy as approximately 80% of RP cases have been found to clinically manifest within 10 months of radiation therapy.^8^ Patient characteristics including age, AJCC stage, smoking history, history of pulmonary disease, radiation treatment details, and systemic therapy characteristics were retrospectively reviewed. Patients with clinical and imaging characteristics consistent with RP were retrospectively assessed for symptom onset, imaging characteristics, and the clinical course of RP. Toxicity grading was based on the Common Terminology Criteria for Adverse Events (CTCAE) v. 5.0 scoring system. This retrospective study was completed under an institutional review board approved protocol.

The subset of consecutive patients treated with platinum‐based cCRT from a previously published stage III NSCLC cohort treated without durvalumab from 2004‐2014 was used as the reference cohort^10^ to which both the onset and rate of RP were compared (Mann‐Whitney U test; significance: *P* ≤ .05). Patients in the reference cohort were similarly also treated with definitive intent thoracic radiation delivered using intensity‐modulated radiation therapy. Among patients in the reference cohort, the median patient age was 63 years, 48% (n = 71) were male, 92% (n = 136) were ever smokers, 50% (n = 74) had an ECOG 0 performance status, and 31% (n = 46) had a prior diagnosis of COPD or Asthma. The median radiation prescription dose among patients in the cCRT alone cohort was 63Gy and the median MLD value was 17Gy (range 4.523). Additionally, 61% (n = 91) were related with a cisplatin containing combination, and 39% (n = 57) were treated with a carboplatin regimen.

### 2.3 Radiation pneumonitis models and analyses

Three existing models for predicting ≥ grade 2 RP were explored. First, the Quantitative Analyses of Normal Tissue Effects in the Clinic (QUANTEC) RP model, which includes MLD only, was evaluated.^8^ The landmark QUANTEC model represents a synthesis of data from a > 70 published studies on RP, most widely uses RP model, and serves as the foundation for other RP models.^8^ Second, the Appelt model was investigated as this model built upon the QUANTEC model and includes six additional patient‐specific variables relating to age, receipt of chemotherapy, preexisting pulmonary comorbidity, smoking status, and tumor location.^9^ Lastly, a recently published model, the Thor model, was assessed as this builds upon the QUANTEC and the Appelt models, but includes only two patient‐specific variables (preexisting pulmonary comorbidity and age).^10^ These three models have been described in detail in their original publications.^8^, ^9^, ^10^ They are briefly summarized in the following: all three models are logistic regression‐based, ie, of type RP = 1/(1 + e^−^ *^x^*), with *x *= −3.9 + (MLD × 0.06) as per the QUANTEC model.^8^ The Appelt model integrates effects of the six aforementioned patient‐specific characteristics with the dose‐response curve from the QUANTEC model as Odds Ratios (ORs; age (cut‐point: 63 years): OR = 1.66; current smoker: OR = 0.62; former smoker: OR = 0.69; inferior/middle tumors: OR = 1.87; preexisting pulmonary comorbidity: OR = 2.27; and sequential as opposed to concurrent chemotherapy: OR = 1.60). The ORs are added multiplicatively, *x*=((4*γ*_50_^0^) × (MLD/D_50_^0^))/ln(OR), and *D*_50_^0^ = 34.4Gy and *γ*_50_^0^ = 1.19. The Thor model is of the same form but here *D*_50_^0^ = 38.8Gy, and *γ*_50_^0^ = 1.01 and the ORs included (the same as in the Appelt model) were only for age and preexisting pulmonary comorbidity.

The three models were evaluated using previously published methods and were tested for their predictive accuracy among patients treated with cCRT plus consolidative durvalumab.^10^ To allow for comparison and to serve as a performance baseline, the three models were also evaluated among patients treated with platinum‐based cCRT alone in the reference cohort. In short, the model assessment approach followed the Transparent Reporting of a multivariate prediction model for Individual Prognosis or Diagnosis (TRIPOD) statement (Type 4 model).^11^ The MLD (lung: total lung volume minus GTV and tumor‐involved lymph nodes) was first converted into the equivalent dose in 2 Gy fractions assuming *α*/*β* = 3 Gy.^8^ Calibration was assessed in quintiles by the Hosmer‐Lemeshow test (*P*_HL_),^12^ and discrimination by the AUC and *P*‐values (from the Spearman's rank, Rs, ordering between the observed and predicted RP rates, *P*_Rs_). A model was deemed appropriate if being significantly predictive (*P*_Rs_ ≤ .05), discriminative (AUC > 0.60), and with *P*_HL_ > .05. The published model coefficients were applied to the corresponding variables in the cohort (*note: no refitting of model coefficients was applied*), and internal generalizability was evaluated over 1000 bootstrap samples (ie, calibration and discrimination measures are reported as the median values across these 1000 samples). An additional analysis was conducted using logistic regression with bootstrapping to determine if PD‐L1 expression either as a continuous variable or categorically as ≥ 50% vs < 50% expression was predictive of ≥ grade 2 RP. All analyses were conducted in MATLAB R2016a (MA, USA).

## 3 RESULTS

### 3.1 Patient and treatment characteristics

Sixty‐two consecutively treated patients with stage III NSCLC treated with definitive‐intent cCRT and at least one dose of consolidative durvalumab between November 2017 and February 2019 were analyzed. Median follow‐up postradiation therapy was 13 months (interquartile range (IQR): 10‐17 months). The median patient age was 66 years, 55% (n = 33) were male, 73% had stage IIIB or IIIC disease, and 53% (n = 33) had an ECOG 0 performance status. In total, 95% (n = 60) were ever smokers, 10% (n = 6) were active smokers during radiation therapy, and 31% (n = 19) had prior diagnosis of COPD or asthma. (Table 1).

**Table 1:** Baseline characteristics

	Patients (n = 62)
Gender	
Female	42% (n = 26)
Male	58% (n = 36)
Age (y)	
Range	49‐86
Median	66
Performance status	
ECOG 0	53% (n = 33)
ECOG 1	47% (n = 29)
Smoking history	
Yes	97% (n = 60)
Smoking during radiation	
Yes	10% (n = 6)
Diabetes mellitus II	
Yes	26% (n = 16)
Pulmonary disease	
COPD	26% (n = 16)
Asthma	5% (n = 3)
Stage	
IIIA	27% (n = 17)
IIIB	53% (n = 33)
IIIC	20% (n = 16)
PD‐L1 expression	
Unavailable	17% (n = 11)
≥1%	53% (n = 33)
≥50%	29% (n = 18)

Patient characteristics are from time of initiation of radiation therapy.

Patients were treated to a median of 60Gy and most patients were treated with concurrent carboplatin/paclitaxel (37%, n = 23) or carboplatin/pemetrexed (31% n = 10). The median MLD was 17Gy (range: 7.2‐21). Patients started their first cycle of durvalumab at a median of 1.5 months after the completion of radiation therapy (Table 2). Thirteen patents (21%) developed ≥ grade 2 any type pneumonitis, a median of 3.3 months (IQR: 2.6‐5.9) after the completion of radiation therapy, and at a median of 1.6 months (IQR: 0.92‐4.71) after the start of consolidative durvalumab. Patients who developed ≥ grade 2 any type pneumonitis were treated with a median of four doses of durvalumab (IQR: 2‐11).

**Table 2:** Chemoradiation treatment characteristics

	Patients (n = 62)
Chemotherapy	
Carboplatin/Paclitaxel	37% (n = 23)
Cisplatin/Pemetrexed	21% (n = 13)
Carboplatin/Pemetrexed	31% (n = 19)
Cisplatin/Etoposide	11% (n = 7)
Radiation Prescription Dose (Gy)	
Range	54‐66
Median	60
Radiation Planning Target Volume (cc)	
Range	90‐1234.4
Median	579
Lung Volume Receiving ≥ 20Gy	
Range	6.4%‐38.5%
Median	30.1%
Mean Lung Dose (Gy)	
Range	7.2‐21.4
Median	17.1

### 3.2 Characterization and clinical course of RP in patients treated with cCRT and durvalumab

Among the 62 patients treated with cCRT and durvalumab, 18% (n = 11) developed ≥ grade 2 RP of which only one patient experienced grade 3 RP. Patients presented with symptoms of progressive dyspnea (n = 11), dry cough (n = 9), and low‐grade fever (n = 1) at a median of 3.4 months (IQR: 2.5‐5.9) after the completion of cCRT and a median of 1.4 months (IQR: 0.9‐4.8) after the start of consolidative durvalumab. CT imaging findings included new or progressive ground‐glass opacities (n = 8) and patchy consolidations (n = 3) involving the radiation field (Table 3). All patients were started on a high‐dose oral prednisone taper (median starting daily dose of 40 mg) and subsequent durvalumab was initially held for all but two patients during the prednisone taper. Four patients had recurrent dyspneic symptoms necessitating repeat steroid taper after completing an initial steroid course and showing symptom improvement. The majority of patients (n = 7) had complete resolution of dyspneic symptoms and no recurrent symptoms > 3 months postcompletion of steroid taper. The remaining patients had improved, but persistent dyspnea above baseline (n = 2), required protracted steroids taper > 6 months (n = 1), or passed away due to disease progression with persistent pneumonitis symptoms (n = 1). All but one patient (n = 10) discontinued further durvalumab therapy (Table 3). Additionally, PD‐L1 expression as a continuous variable and categorially as PD‐L1 ≥ 50% did not predict for ≥ grade 2 RP (*P* = .57 and *P* = .49, respectively).

**Table 3:** Characterization of radiation pneumonitis

Patient & treatment characteristics	Clinical presentation	Imaging findings	Pneumonitis treatment course
59‐y‐old former smoker with stage IIIC NSCLC of RLL completed 60Gy RT concurrent with cisplatin/etoposide followed by initiation of durvalumab 7 wks post‐RT Mean lung dose: 21.3 Gy Lung volume ≥ 20 Gy: 37.1%	Increased dyspnea and new progressive dry cough 2.8 mo after RT end	Patchy ground‐glass changes within RT field	Started on Prednisone 40 mg daily with 8‐wk taper. Durvalumab discontinued. Respiratory symptoms improved within 1 wk. No recurrent symptoms 6 mo poststeroids
80‐y‐old former smoker with stage IIIB NSCLC of RUL completed 60Gy RT concurrent with carboplatin/pemetrexed followed by initiation of durvalumab 3.1 wks post‐RT Mean lung dose: 18.2 Gy Lung volume ≥ 20 Gy: 31.2%	Increased dyspnea 3.6 mo after RT end	Patchy ground‐glass opacities within RT field	Started on Prednisone 30 mg daily tapered over 18 wks. Durvalumab not held. Respiratory symptoms improved to baseline. No recurrent symptoms 3 mo poststeroids
56‐y‐old former smoker with stage IIIA NSCLC of RUL completed 60Gy RT concurrent with cisplatin/etoposide followed by initiation of durvalumab 2.4 wks post‐RT Mean lung dose: 15.6 Gy Lung volume ≥ 20 Gy: 27.3%	Progressive dry cough, dyspnea with low‐grade fever 9.5 mo after RT end	Increased patchy consolidations within RT field	Durvalumab held and short‐course steroids trialed. Symptoms returned once steroids stopped. Then, started on 40 mg daily Prednisone with 8‐wk taper with symptoms resolution. Symptoms returned within 3 mo and Prednisone restarted with improvement in symptoms. Durvalumab discontinued
86‐y old former smoker with stage IIIA NSCLC of LUL completed 60Gy RT concurrent with carboplatin/paclitaxel followed by initiation of durvalumab 7 wks post RT Mean lung dose: 17.5 Gy Lung volume ≥ 20 Gy: 30.1%	Progressive dry cough and persistent dyspnea 3.3 mo after RT end	Increased patchy consolidation within RT field	Started on Prednisone 50 mg taper. Cough improved within 1 wk. Durvalumab held and discontinued. Dyspnea worsened after completion of Prednisone taper. Restarted Prednisone and continues 9 mo post initial presentation with slowly improving dyspnea.
71‐y‐old current smoker with stage IIIA NSCLC of LUL completed 60Gy RT concurrent with carboplatin/paclitaxel followed by initiation of durvalumab 5.6 wks post RT Mean lung dose: 14.4 Gy Lung volume ≥ 20 Gy: 27.6%	Progressive dry cough and dyspnea 5.9 mo after RT end	Increased ground‐glass opacities within RT field	Durvalumab held and discontinued. Steroids not initially started given DMII, but cough progressed. Started on Prednisone taper with symptoms improvement. No recurrent symptoms and back to near baseline respiratory status 6 mo after initial presentation
74‐y‐old former smoker with stage IIIC NSCLC of RUL completed 66Gy RT concurrent with carboplatin/paclitaxel followed by initiation of durvalumab 3.6 wks post RT Mean lung dose: 16.2 Gy Lung volume ≥ 20 Gy: 22.6%	Progressive dry cough and dyspnea 5.7 mo after RT end	Increased patchy consolidations within RT field	Started on Prednisone 40 mg daily with taper. Durvalumab held and discontinued. Symptoms improved within 1 wk. No recurrent symptoms 3 mo poststeroids
61‐y‐old former smoker with stage IIIB NSCLC of LUL completed 60Gy RT concurrent with carboplatin/paclitaxel followed by initiation of durvalumab 7.8 wks post‐RT Mean lung dose: 10.2 Gy Lung volume ≥ 20 Gy: 14.6%	Progressive new dyspnea 2.4 mo after RT end	Increased ground‐glass opacities within RT field	Prednisone 40 mg daily started with taper. Durvalumab continued during Prednisone. Symptoms improved within 1 wk of steroid initiation. Symptoms returned after completion of Prednisone taper, Prednisone restarted, and tapered over 4 wks. Durvalumab discontinued. No recurrent symptoms 3 mo poststeroids
75‐y‐old former smoker with stage IIIB NSCLC of RUL completed 60Gy RT concurrent with carboplatin/paclitaxel followed by initiation of durvalumab 6.1 wks post‐RT Mean lung dose: 17.9 Gy Lung volume ≥ 20 Gy: 36.7%	Progressive dry cough and dyspnea with minimal exertion 2.76 mo after RT end	New patchy ground‐glass opacities within RT field	Initially hospitalized for cough and dyspnea and discharged on Prednisone 60 mg daily with planned taper. Durvalumab held and discontinued. Dyspnea worsened 4 wks into taper at 20 mg daily. Prednisone increased, and taper extended for 3 mo with symptom improvement, but with persistent dyspnea greater than baseline. Patient passed away because of disease progression.
66‐y‐old former smoker with stage IIIA NSCLC of RUL completed 60Gy RT concurrent with cisplatin/pemetrexed followed by initiation of durvalumab 9 wks post‐RT Mean lung dose: 19.8 Gy Lung volume ≥ 20 Gy: 34.6%	Progressive dry cough, and increased dyspnea 8.9 mo after RT end	New nodular ground‐glass opacities within RT field	Started Prednisone taper with improvement in cough and dyspnea. Durvalumab held and discontinued. Referred to pulmonology for evaluation of residual dyspnea above baseline
61‐year‐old never smoker with stage IIIB NSCLC of RUL completed 60Gy RT concurrent with carboplatin/pemetrexed followed by initiation of durvalumab 8.2 wks post‐RT Mean lung dose: 17.7 Gy Lung volume ≥ 20 Gy: 28.5%	Progressive dry cough, dyspnea with minimal exertion 2.4 mo after RT end	Increase ground‐glass opacities and patchy consolidations within RT field	Durvalumab held. Started on Prednisone 60 mg daily with taper. Symptoms improved in 2 wks and back to baseline respiratory function at end of taper. Symptoms returned within 1 mo after steroids stopped. Prednisone restarted and tapered over 3 mo. Durvalumab discontinued given disease progression. Now follows with pulmonology, no worsening pulmonary symptoms 6 mo poststeroids
72‐y old former smoker with stage IIIC NSCLC of LUL completed 60Gy RT concurrent with carboplatin/pemetrexed followed by initiation of durvalumab 5.9 wks post RT Mean lung dose: 17.7 Gy Lung volume ≥ 20 Gy: 28.5%	Increased cough and dyspnea 5.9 mo after RT end	Increased ground‐glass opacities within RT field	Durvalumab held and discontinued. Started on Prednisone taper, then hospitalized for pneumonia and treated with methylprednisolone, and discharged with Prednisone taper. No recurrent symptoms 6 mo poststeroids

**Figure 1:**
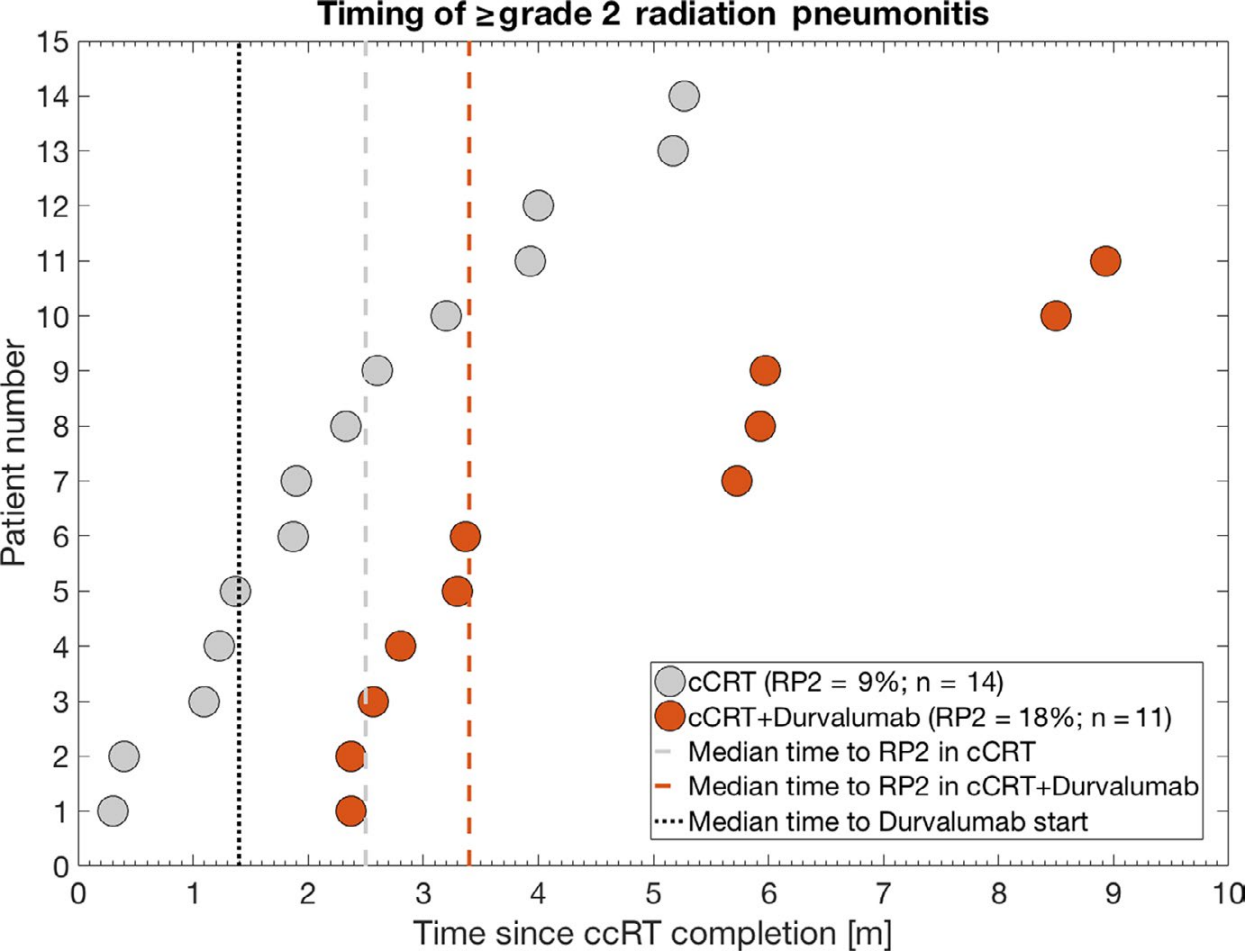
Time to ≥ grade 2 radiation pneumonitis (RP2) in the concurrent chemoradiation alone cohort (cCRT, grey circles) and in the concurrent chemoradiation and consolidative durvalumab cohort (cCRT + durvalumab cohort, orange circles) as well as their cohort median time to RP2 (dashed grey and dashed orange lines, respectively). *Note: The cohort median time to Durvalumab start has been inserted as well (black dotted line)*

Compared to the reference cohort treated with cCRT alone, the time to RP onset was significantly longer in patients treated with consolidative durvalumab (median time to onset: 3.4 months vs 2.1 months (*P* = .01; Figure 1)). Additionally, the frequency of RP was numerically greater in patients treated with consolidative durvalumab vs the reference cohort, although this finding did not reach statistical significance in this analysis (18% vs 9%; *P* = .09).

### 3.3 Assessing the validity of existing RP models in patients treated with cCRT and durvalumab

**Figure 2:**
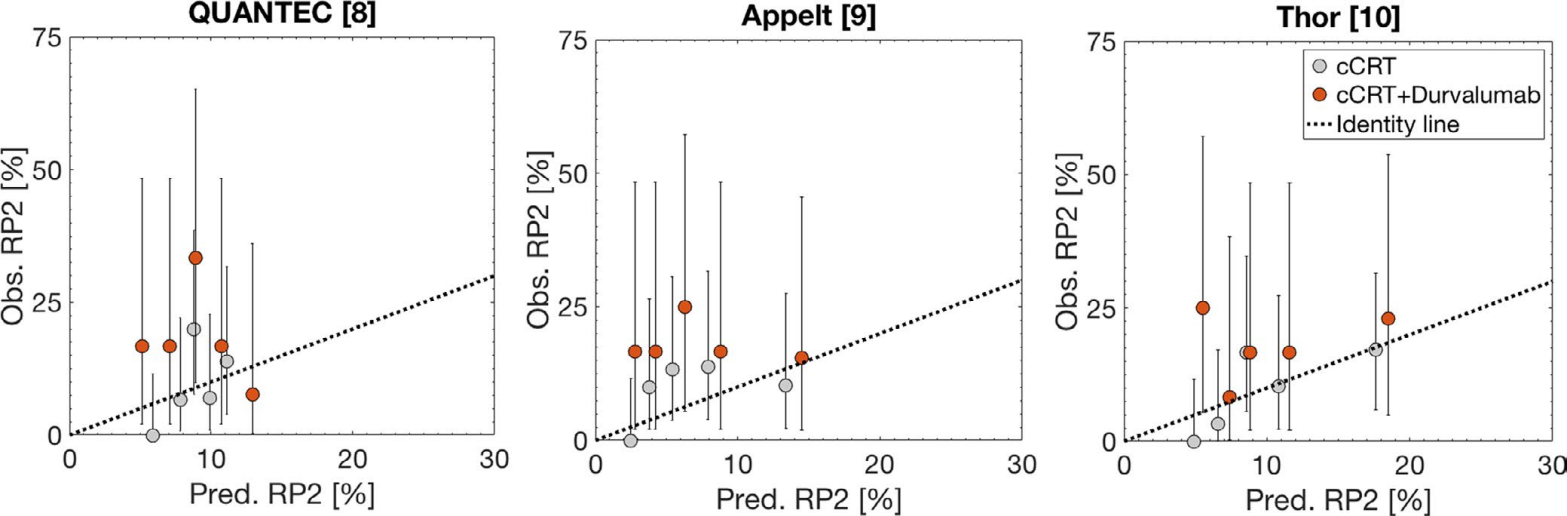
Assessment of the QUANTEC, Appelt, and Thor models in predicting ≥ grade 2 radiation pneumonitis (RP2) in patients treated with concurrent chemoradiation alone cohort (cCRT, grey quintiles) and patients treated with concurrent chemoradiation and consolidative durvalumab (cCRT + durvalumab, orange quintiles). *Note: Black dotted identity lines have been inserted to reflect over/underestimation of RP2 prediction by each model*

Predictions of ≥ grade 2 RP from all three models^9^ were unsuccessful in explaining the observed rate of ≥ grade 2 RP in patients treated with cCRT and consolidative durvalumab (AUC = 0.50; *P*_Rs_ = .42‐.52; *P*_HL_ = .98‐1.00; Figure 2). Uniformly, the predicted rate of RP from these models significantly underestimated the observed RP rate.

In contrast, the prediction of RP among patients in the reference cohort treated with cCRT alone better agreed with the observed rates (AUC = 0.63‐0.74; *P* = .004‐0.16; *P*_HL_ = .60‐.91; Figure 2). In this cohort, the Thor model had the overall best performance and significantly predicted RP (AUC = 0.74; *P*_Rs_ = .004; *P*_HL_ = .60).

## 4 DISCUSSION

We present the largest detailed characterization to date of the onset and clinical course of RP among patients treated cCRT and durvalumab and also evaluated the validity of published RP models in predicting for symptomatic RP with this new standard of care. Symptomatic RP occurred in 18% of patients treated with cCRT and durvalumab vs 9% of patients treated with cCRT alone. We also found the onset of RP symptoms to be significantly later in the patients treated with cCRT and durvalumab. The majority of RP cases responded to high‐dose oral glucocorticoid therapy, and most patients discontinued durvalumab with no recurrent dyspneic symptoms. Validated toxicity models found to be predictive for symptomatic RP in the cCRT alone era were not effective in predicting for symptomatic RP in the patients treated with cCRT and consolidative durvalumab, and uniformly underestimated the observed RP rates.

Radiation pneumonitis is a potentially life‐threatening, dose‐limiting toxicity of thoracic radiation.^13^ The characteristic symptoms of RP remain nonspecific, but include shortness of breath, cough, and low‐grade fever occurring weeks to several months after the completion of radiation.^14^, ^15^, ^16^ Radiographic changes have been classically noted predominantly within, but not limited to the radiation field.^17^, ^18^, ^19^ The incidence of symptomatic RP in patients with lung cancer treated with thoracic radiation is estimated to be 10%‐40%^20^ with the incidence of fatal pneumonitis in NSCLC patients treated with concurrent chemoradiation estimated to be < 2%.^2^ Any grade pneumonitis due to anti‐PD‐1/PD‐L1‐directed therapies has been estimated to occur in 4% of NSCLC patients, with higher‐grade pneumonitis estimated to occur in < 2% of patients.^21^, ^22^ The onset of pneumonitis symptoms in patients treated with anti‐PD‐1/PD‐L1 therapies has been found to be variable, ranging from days to over a year after treatment initiation with the median time to onset found to be 2.8 months in a prior study.^22^ Prior studies have also found radiologic features to be diverse, with no specific pathognomonic feature attributed to anti‐PD‐1/PD‐L1 pneumonitis.^22^

Clinical data from prospective trials, although limited, suggest that thoracic radiation therapy may potentially increase pulmonary toxicities of concurrent or sequential anti‐PD‐1/PD‐L1 therapies in NSCLC.^6^, ^7^ These data suggest mechanisms of interaction between thoracic RT and anti‐PD‐L1 therapies that can potentially impact the development of pneumonitis. The PACIFIC trial, which lead to the approval of durvalumab after cCRT in stage III NSCLC, found that the risk of developing any grade, any type pneumonitis was higher in patients treated with durvalumab vs placebo (33.9% vs 24.8%).^3^ Additionally, any grade RP in specific was found in more patients treated with consolidative durvalumab vs placebo (20% vs 15.8%), and grade 3 or 4 RP was also found in more patients treated with durvalumab (1.5% vs 0.4%).^1^ Our finding of 18% of patients treated with cCRT and durvalumab to develop ≥ grade 2 RP appears congruent with the PACIFIC trial data. We also found numerically greater cases of symptomatic RP in patients treated with consolidative durvalumab vs our reference cohort treated with cCRT alone (18% vs 9%). The median time to RP onset was significantly longer in patients treated with durvalumab than in patients treated with cCRT alone which also suggests interactions between these therapies that modify the risk of developing RP. Reassuringly, the clinical course of RP among patients treated with consolidative durvalumab appears no worse, as most patients were successfully treated with a course of oral glucocorticoids with symptom resolution or improvement.^14^

Multiple factors including mean lung radiation dose (MLD), smoking history, chemotherapy exposure, and preexisting pulmonary disease have been found to impact the risk of symptomatic RP.^2^, ^8^, ^9^, ^23^, ^24^, ^25^, ^26^ Biologic factors, including circulating levels of transforming growth factor β and interleukin 6 have also been found to be predictive of RP.^27^, ^28^ The plurality of data indicates that the risk of RP is increased with increased radiation dose and irradiated lung volume.^20^, ^29^, ^30^, ^31^ Therefore, multiple radiation dose parameters are used in clinical practice to optimize radiation planning to reduce the risk of RP. Furthermore, despite prophylactic corticosteroids during radiotherapy being found to reduce the risk of RP, no preventative therapeutic strategy is standardly employed in practice, therefore, radiation plan optimization remains essential to mitigating the risk of RP.^32^ However, all three RP models tested, which all incorporate MLD, were widely inadequate in predicting symptomatic RP in patients treated with cCRT and durvalumab. This finding is supported by data from the ongoing phase‐II NICOLAS trial in which chemoradiation is combined with upfront concurrent anti‐PD‐1 therapy and consolidative anti‐PD‐1 therapy.^33^ Based on their interim analysis, ‘standard’ variables such as the MLD and the volume of lung receiving 20Gy were not found to be associated with pneumonitis incidence of any grade.^33^ These building data support the need to reexamine thoracic radiation planning in the context of patients receiving consolidative anti‐PD‐L1 therapy to determine variables that can better optimize treatment planning. Given the identified limitations of currently used planning parameters, clinicians should strive for radiation plans with the lowest reasonable impact on thoracic organs, even if plans meet currently accepted constraints.

The interpretation of this study is limited by its retrospective nature and the inclusion of a single high‐volume tertiary cancer center. Our patients were also mostly stage IIIB and IIIC, in line with our institutional standard for surgery for low‐volume N2 disease, which could have impacted the incidence of RP. However, we used an institutional reference cohort to best allow for comparisons between treatment eras. Defining the diagnosis of RP is a known challenge given its nonspecific nature ^16^ and, therefore, can result in bias. Additionally, the analysis of the timing of RP onset is limited by the patients who are excluded from durvalumab initiation due to preexisting RP, and the heightened follow‐up among patients treated with durvalumab given the q2week dosing schedule. As more patients are treated with this new standard of care, future studies examining these findings in a larger cohort of patients and better identifying predictors of radiation pneumonitis are necessary. However, our data provide a much needed updated assessment of RP that can help optimize the management of patients with stage III NSCLC treated concurrent chemoradiation and durvalumab.

## CONFLICTS OF INTEREST

Narek Shaverdian: No COI to report. Maria Thor: No COI to report. Annemarie F. Shepard: Reports honoraria from ASCO. Michael D. Offin: Reports advisory role for PharMar, Novartis, and Targeted Oncology. Reports honoraria from Bristol‐Myers Squibb and Merck Sharp & Dohme. Andrew Jackson: Reports grants from NIH/NCI, outside of the submitted work. Abraham J. Wu: Reports research support from CivaTech Oncology, Inc, nonfinancial support from AlphaTau Medical, personal fees from MoreHealth, and personal fees from AstraZeneca. Daphna Y. Gelblum: No COI to report. Ellen D. Yorke: No COI to report. Charles B. Simone II: Reports honoraria from Varian Medical Systems. Jamie E. Chaft: Reports both research funding and consulting roles with Bristol‐Myers Squibb, Merck, Genentech, and AstraZeneca. Mathew D. Hellmann: Reports personal fees from Genentech, grants, personal fees, and nonfinancial support from Bristol Myers Squibb, personal fees from Merck, personal fees and nonfinancial support from AstraZeneca, personal fees from Mirati, personal fees from Syndax, personal fees and equity from Shattuck Labs, personal fees and equity from Immunai, personal fees from Nektar, and personal fees from Blueprint Medicines. In addition, Dr Hellmann has a pending patent Determinants of Cancer Response to Immunotherapy by PD‐1 Blockade (PCT/US2015/062208) licensed to PGDx. Daniel R. Gomez: Reports honoraria from Merck, BMS, AstraZeneca, Reflexion, Medscape, Vindico, US Oncology, and Varian. Reports research support from Merck, BMS, AstraZeneca, and Varian. Serves on advisory board for AstraZeneca. Andreas Rimner: Reports grants from Varian Medical Systems, grants from Boehringer Ingelheim, grants from Pfizer, grants and personal fees from AstraZeneca, grants and personal fees from Merck, personal fees from Research to Practice, personal fees from Cybrexa, nonfinancial support from Philips/Elekta, and personal fees from MoreHealth. Joseph O. Deasy: No COI to report.

## AUTHOR CONTRIBUTION

Narek Shaverdian: conceptualization, data collection, data analysis, and writing^.^ Maria Thor: conceptualization, data collection, data analysis, and writing. Annemarie F. Shepherd: writing and editing. Michael D. Offin: methodology, writing, and editing^.^ Andrew Jackson: conceptualization, analysis, methodology, and editing. Abraham J. Wu: Methodology and editing. Daphna Y. Gelblum: Editing. Ellen D. Yorke: writing and editing. Charles B. Simone, II: methodology, analysis, and writing. Jamie E. Chaft: Analysis and editing. Matthew D. Hellmann: methodology, analysis, and editing. Daniel R. Gomez: Conceptualization and editing. Andreas Rimner: data collection, analysis, writing, and editing. Joseph O. Deasy: Conceptualization, analysis, writing, and editing.

## Data Availability

The data that support the findings of this study are available from the corresponding author upon reasonable request.
